# A Comparison of Statistical Methods to Construct Confidence Intervals and Fiducial Intervals for Measures of Health Disparities

**DOI:** 10.3390/ijerph21020208

**Published:** 2024-02-10

**Authors:** Tengfei Li, Anca D. Dragomir, George Luta

**Affiliations:** 1 Department of Biostatistics, Bioinformatics and Biomathematics, Georgetown University, Washington, DC 20057, USA; tl602@georgetown.edu; 2 Department of Oncology, Georgetown University, Washington, DC 20057, USA; anca.dragomir@georgetown.edu

**Keywords:** delta method, Monte Carlo simulation, fiducial inference, confidence interval, fiducial interval, measures of health disparities

## Abstract

Health disparities are differences in health status across different socioeconomic groups. Classical methods, e.g., the Delta method, have been used to estimate the standard errors of estimated measures of health disparities and to construct confidence intervals for these measures. However, the confidence intervals constructed using the classical methods do not have good coverage properties for situations involving sparse data. In this article, we introduce three new methods to construct fiducial intervals for measures of health disparities based on approximate fiducial quantities. Through a comprehensive simulation study, We compare the empirical coverage properties of the proposed fiducial intervals against two Monte Carlo simulation-based methods—utilizing either a truncated Normal distribution or the Gamma distribution—as well as the classical method. The findings of the simulation study advocate for the adoption of the Monte Carlo simulation-based method with the Gamma distribution when a unified approach is sought for all health disparity measures.

## 1. Introduction

In recent years, more and more attention has been given to health equity, one of the goals of the Healthy People 2020 [1]. The World Health Organization (WHO) has pointed out the “gap” in health between segments of the population [2]. A health disparity is defined as “a particular type of health difference that is closely linked with social, economic, and/or environmental disadvantage” [1]. The US National Institute on Minority Health and Health Disparities has raised national awareness about the prevalence and impact of health disparities that would adversely affect groups of people who are more vulnerable to health-related issues. Last but not least, the US Centers for Disease Control and Prevention (CDC) has played an important role in identifying the factors that lead to health disparities among racial, ethnic, geographic, and other socioeconomic groups, an example being the 2011 CDC Health Disparities and Inequalities Report [3].

There are multiple measures available to quantify the presence of health disparities across socioeconomic groups. The Health Disparity Calculator (HD*Calc), version 2.0.0, is a free statistical software that calculates estimates of commonly used measures of health disparities and constructs corresponding confidence intervals (CIs), using both classical and Monte Carlo simulation (MCS)-based methods [4,5,6]. The measures implemented in HD*Calc belong to three categories: absolute measures, relative measures and pairwise comparison measures. The absolute measures include range difference (RD), between-group variance (BGV), extended absolute concentration index (eACI) and the slope index of inequality (SII). The relative measures include range ratio (RR), index of disparity (IDisp), mean log deviation (MLD), Theil’s index (T), extended relative concentration index (eRCI), relative index of inequality (RII) and the Kunst–Mackenbach relative index (KMI). The pair comparison methods include pair difference (PD) and pair ratio (PR). Although HD*Calc was designed to analyze data from the Surveillance, Epidemiology, and End Results (SEER) Program, the software can also be used for other population-based health data.

Two articles have formally evaluated the empirical coverage properties of the methods to construct CIs implemented in HD*Calc. The first article has compared the classical method and the MCS-based method using the truncated Normal distribution [7]. The authors concluded that the two methods work well except for situations when the data are sparse. As a general solution to dealing with sparse data, the second article has proposed the use of the MCS-based method with the Gamma distribution [8]. The MCS-based method with the Gamma distribution is currently the recommended approach to construct CIs for the measures of health disparities implemented in HD*Calc. By extending the work from Krishnamoorthy and Lee 2010 [9] to the case of measures of health disparities, the aims of the current article are to introduce three new methods to construct fiducial intervals for measures of health disparities, based on approximate fiducial quantities, and to compare their frequentist properties, i.e., their empirical coverage performance, with those of existing methods by using a simulation study involving nine different scenarios that allow different combinations of sample sizes and true rates per cell (where the cells are all cross-classifications of age groups and socioeconomic groups).

This paper is organized as follows. We review the measures of health disparities implemented in HD*Calc and describe the statistical methods used to construct confidence intervals and fiducial intervals, including the classical method, the MCS-based methods and the proposed new fiducial methods. We describe the simulation study used to evaluate the empirical coverage performance of the intervals constructed using these methods, and, at the end, report and discuss the results of the simulation study. We provide all the results of the simulation study in Appendix A.

## 2. Materials and Methods

### 2.1. Background and Notation

In what follows, in contrast to previous work [6,10], we clearly distinguish between functions of parameters and their estimates. We denote by $\lambda_{jk}$ the true rate (e.g., cancer rate) of the *k*-th age group within the *j*-th socioeconomic group, j=1,…,J,k=1,…,K. The true age-adjusted rate of the *j*-th socioeconomic group, $\mu_{j}$, is defined as

(1) $$\begin{array}{c} \mu_{j}=\sum_{k=1}^{K}w_{k}\lambda_{jk}, \end{array}$$

where $w_{k}$ is the weight for the *k*-th age group within the *j*-th socioeconomic group.

To estimate the true rate $\lambda_{jk}$ and the true age-adjusted rate $\mu_{j}$, we use the unbiased estimators $R_{jk}$ and $Y_{j}$, respectively. We denote the estimated rate of the *k*-th age group within the *j*-th socioeconomic group as

$$R_{jk}=\frac{D_{jk}}{n_{jk}},\;j=1,\ldots ,J,\;k=1,\ldots ,K,$$

where $D_{jk}$ denotes the number of events and $n_{jk}$ denotes the number of persons (or person-years). The estimated age-adjusted rate is

(2) $$\begin{array}{c} Y_{j}=\sum_{k=1}^{K}w_{k}R_{jk}. \end{array}$$

We assume that $D_{jk}∼\mathrm{Poisson}\left(n_{jk}\lambda_{jk}\right)$. The variance of the estimator $Y_{j}$ is

(3) $$\begin{array}{cc} \sigma_{j}^{2} & =\mathrm{Var}\left(Y_{j}\right)\\ \; & =\sum_{k=1}^{K}w_{k}^{2}\mathrm{Var}\left(R_{jk}\right)\\ \; & =\sum_{k=1}^{K}\frac{w_{k}^{2}}{n_{jk}^{2}}\mathrm{Var}\left(D_{jk}\right)\\ \; & =\sum_{k=1}^{K}\frac{w_{k}^{2}}{n_{jk}^{2}}n_{jk}\lambda_{jk}\\ \; & =\sum_{k=1}^{K}\frac{w_{k}^{2}}{n_{jk}}\lambda_{jk}. \end{array}$$

An unbiased estimate of this variance is

(4) $${\hat{\sigma }}_{j}^{2}=\sum_{k=1}^{K}\frac{w_{k}^{2}}{n_{jk}^{2}}D_{jk}.$$

We define the vector of *J* estimators/estimates as $Y=\left(Y_{1},\ldots ,Y_{J}\right)$ and the vector of the *J* true age-adjusted rates as $\mu =\left(\mu_{1},\ldots ,\mu_{J}\right)$, where $E\left(Y_{j}\right)=\mu_{j}$ for j=1,…,J. In what follows, we assume that the *J* estimators are independent and the estimated variances of these estimators are given by ${\hat{\sigma }}_{j}^{2}$ for j=1,…,J.

### 2.2. Measures of Health Disparities

The (true) measures of health disparities are functions of parameters, F(μ), although they are often not clearly distinguished from their estimates, F(Y) which may lead to confusion. Depending on the function F(·), we obtain different measures of health disparities. In what follows, we will present the measures implemented in HD*Calc. For simplicity, we will refer to the (true) age-adjusted rates simply as (true) rates.

#### 2.2.1. Range Difference (RD) and Pair Difference (PD)

The range difference is the difference between the true rates of the best and the worst socioeconomic groups

(5) $$\begin{array}{c} \mathrm{RD}=\mu_{\max}-\mu_{\min}, \end{array}$$

where $\mu_{\max}=\max_{j}\mu_{j}$ and $\mu_{\min}=\min_{j}\mu_{j}$. It is estimated by

(6) $$\begin{array}{c} \hat{\mathrm{RD}}=Y_{\left(J\right)}-Y_{\left(1\right)}, \end{array}$$

where $Y_{\left(j\right)}$ is the *j*-th order statistic of the observed values of *Y*. This may cause problems as $Y_{\left(J\right)}$ and $Y_{\left(1\right)}$ may not necessarily be unbiased estimators of $\mu_{\max}$ and $\mu_{\min}$, respectively. To address this issue, we may fix in advance the groups to be compared and consider instead the pair difference

(7) $$\begin{array}{c} \mathrm{PD}=\mu_{1}-\mu_{2}, \end{array}$$

which has as its estimator

(8) $$\begin{array}{c} \hat{\mathrm{PD}}=Y_{1}-Y_{2}, \end{array}$$

where $Y_{1}$ and $Y_{2}$ are estimators of $\mu_{1}$ and $\mu_{2}$, respectively.

#### 2.2.2. Between-Group Variance (BGV)

BGV is calculated using the squares of the differences between the socioeconomic groups’ rates and the population mean rate, with weighting by the corresponding population share

(9) $$\begin{array}{c} \mathrm{BGV}=\sum_{j=1}^{J}p_{j}{\left(\mu_{j}-\bar{\mu }\right)}^{2}, \end{array}$$

where

(10) $$\begin{array}{c} p_{j}=\frac{n_{j}}{\sum_{s=1}^{J}n_{s}} \end{array}$$

is the population share of the *j*-th socioeconomic group (treated as essentially known, i.e., estimated with negligible sampling error), and

(11) $$\begin{array}{c} \bar{\mu }=\sum_{j=1}^{J}p_{j}\mu_{j} \end{array}$$

is the population mean rate. The estimator of BGV is given by

(12) $$\begin{array}{c} \hat{\mathrm{BGV}}=\sum_{j=1}^{J}p_{j}{\left(Y_{j}-\bar{Y}\right)}^{2}, \end{array}$$

where $\bar{Y}=\sum_{j=1}^{J}p_{j}Y_{j}$.

#### 2.2.3. Range Ratio (RR) and Pair Ratio (PR)

The RR is similar to the RD, where we replace the subtraction with division. It is defined as

(13) $$\begin{array}{c} \mathrm{RR}=\frac{\mu_{\max}}{\mu_{\min}}, \end{array}$$

where $\mu_{\max}$ and $\mu_{\min}$ are defined in (5). It is estimated by

(14) $$\begin{array}{c} \hat{\mathrm{RR}}=\frac{Y_{\left(J\right)}}{Y_{\left(1\right)}}, \end{array}$$

where $Y_{\left(1\right)}$ and $Y_{\left(J\right)}$ are defined in (6).

Similarly to PD, PR is defined as

(15) $$\begin{array}{c} \mathrm{PR}=\frac{\mu_{1}}{\mu_{2}}, \end{array}$$

and is estimated by

(16) $$\begin{array}{c} \hat{\mathrm{PR}}=\frac{Y_{1}}{Y_{2}}, \end{array}$$

where $Y_{1}$ and $Y_{2}$ are estimators of $\mu_{1}$ and $\mu_{2}$, respectively.

#### 2.2.4. Relative Concentration Index (RCI) and Extended Relative Concentration Index (eRCI)

RCI is a measure that can be used only with ordinal socioeconomic groups. It is defined by Kakwani et al., 1997 [11] as

(17) $$\begin{array}{c} \mathrm{RCI}=\frac{2}{\bar{\mu }}\left(\sum_{j=1}^{J}p_{j}z_{j}\mu_{j}\right)-1, \end{array}$$

where $\bar{\mu }$ and $p_{j}$ are defined in (11) and (10), respectively. Here, $z_{j}$ is the relative rank of the *j*-th ordinal socioeconomic group, defined as

(18) $$\begin{array}{c} z_{j}=\sum_{k=1}^{j}p_{k}-\frac{1}{2}p_{j}. \end{array}$$

RCI is estimated by

(19) $$\begin{array}{c} \hat{\mathrm{RCI}}=\frac{2}{\bar{Y}}\left(\sum_{j=1}^{J}p_{j}z_{j}Y_{j}\right)-1, \end{array}$$

where $\bar{Y}$, $p_{j}$ and $z_{j}$ are defined in (12), (10) and (18), respectively.

Yu et al., 2019 [12] used eRCI as a measure of health disparities. It can be calculated as

(20) $$\begin{array}{c} \mathrm{eRCI}=\nu \sum_{j=1}^{J}p_{j}{\left(1-z_{j}\right)}^{\nu -1}-\frac{\nu }{\bar{\mu }}\sum_{j=1}^{J}p_{j}\mu_{j}{\left(1-z_{j}\right)}^{\nu -1}, \end{array}$$

where ν>0 is the aversion parameter, and $\mu_{j}$, $\bar{\mu }$, $p_{j}$ and $z_{j}$ are the same as in (17). The estimator is

(21) $$\begin{array}{c} \hat{\mathrm{eRCI}}=\nu \sum_{j=1}^{J}p_{j}{\left(1-z_{j}\right)}^{\nu -1}-\frac{\nu }{\bar{Y}}\sum_{j=1}^{J}p_{j}Y_{j}{\left(1-z_{j}\right)}^{\nu -1}. \end{array}$$

If ν=2 we obtain RCI. In this article, we use ν=3 for eRCI.

#### 2.2.5. Absolute Concentration Index (ACI) and Extended Absolute Concentration Index (eACI)

ACI is the absolute version of RCI. It has the following formula

(22) $$\begin{array}{c} \mathrm{ACI}=\bar{\mu }\;\mathrm{RCI}=\sum_{j=1}^{J}p_{j}\left(2z_{j}-1\right)\mu_{j}, \end{array}$$

which can be estimated by

(23) $$\begin{array}{c} \hat{\mathrm{ACI}}=\sum_{j=1}^{J}p_{j}\left(2z_{j}-1\right)Y_{j}, \end{array}$$

where $p_{j}$ and $z_{j}$ are defined in (10) and (18), respectively.

Yu et al. 2019 [12] used eACI as a measure of health disparities. It can be calculated as

(24) $$\begin{array}{c} \mathrm{eACI}=\bar{\mu }\;\mathrm{eRCI}=\nu \bar{\mu }\sum_{j=1}^{J}p_{j}{\left(1-z_{j}\right)}^{\nu -1}-\nu \sum_{j=1}^{J}p_{j}\mu_{j}{\left(1-z_{j}\right)}^{\nu -1}, \end{array}$$

where ν>0 is the aversion parameter, and $\mu_{j}$, $\bar{\mu }$, $p_{j}$ and $z_{j}$ are the same as in (22). The estimator is

(25) $$\begin{array}{c} \hat{\mathrm{eACI}}=\nu \bar{Y}\sum_{j=1}^{J}p_{j}{\left(1-z_{j}\right)}^{\nu -1}-\nu \sum_{j=1}^{J}p_{j}Y_{j}{\left(1-z_{j}\right)}^{\nu -1}. \end{array}$$

If ν=2 we obtain ACI. In this article, we use ν=3 for eACI.

#### 2.2.6. Slope Index of Inequality (SII)

SII measures the difference in rates between a hypothetical person with $z_{j}=1$ and a hypothetical person with $z_{j}=0$. It was introduced by Preston, Haines and Pamuk, 1981 [13] using a simple linear regression model

(26) $$\begin{array}{c} E\left(Y_{j}|z_{j}\right)=\beta_{0}+\beta_{1}z_{j}, \end{array}$$

where $z_{j}$ is defined in (18) and SII = $\beta_{1}$.

Since the regression is run on grouped data, SII is estimated using the least squares weighted by the population shares $p_{j}$

(27) $$\begin{array}{c} \hat{\mathrm{SII}}=\frac{\sum_{j}\left(p_{j}z_{j}Y_{j}\right)-\sum_{j}\left(p_{j}z_{j}\right)\sum_{j}\left(p_{j}Y_{j}\right)}{\sum_{j}\left(p_{j}z_{j}^{2}\right)-{\left(\sum_{j}\left(p_{j}z_{j}\right)\right)}^{2}}, \end{array}$$

where $p_{j}$ is defined in (10).

#### 2.2.7. Index of Disparity (IDisp)

The index of disparity (IDisp) measures the relative difference between the rates of the socioeconomic groups and a reference rate as a proportion of the reference rate. It was first introduced by Pearcy and Keppel, 2002 [14] as

(28) $$\begin{array}{c} {\mathrm{IDisp}}_{\mathrm{PK}}=\frac{1}{J}\sum_{j=1}^{J}\frac{|\mu_{j}-\bar{\mu }|}{\bar{\mu }}\times 100. \end{array}$$

A version of IDisp is replacing the population mean rate, $\bar{\mu }$, with the rate of a reference group, $\mu_{\mathrm{ref}}$, which is

(29) $$\begin{array}{c} \mathrm{IDisp}=\frac{1}{J-1}\sum_{j=1,j\neq \mathrm{ref}}^{J}\frac{|\mu_{j}-\mu_{\mathrm{ref}}|}{\mu_{\mathrm{ref}}}\times 100, \end{array}$$

The corresponding estimator is

(30) $$\begin{array}{c} \hat{\mathrm{IDisp}}=\frac{1}{J-1}\sum_{j=1,j\neq \mathrm{ref}}^{J}\frac{|Y_{j}-Y_{\mathrm{ref}}|}{Y_{\mathrm{ref}}}\times 100, \end{array}$$

where $Y_{\mathrm{ref}}$ is the estimator of $\mu_{\mathrm{ref}}$. To eliminate the absolute values from the formula, HD*Calc recommends the use of the group with the smallest rate as the reference group.

#### 2.2.8. Mean Log Deviation (MLD)

MLD is defined as

(31) $$\begin{array}{cc} \mathrm{MLD} & =\sum_{j=1}^{J}p_{j}\left(-\log\gamma_{j}\right)\\ \; & =\log\left(\bar{\mu }\right)-\sum_{j=1}^{J}p_{j}\log\left(\mu_{j}\right), \end{array}$$

where $\bar{\mu }$ and $p_{j}$ are defined in (11) and (10), respectively, and

(32) $$\begin{array}{c} \gamma_{j}=\frac{\mu_{j}}{\bar{\mu }} \end{array}$$

is the ratio of the rate of the *j*-th socioeconomic group and the population mean rate. It is estimated by

(33) $$\begin{array}{cc} \hat{\mathrm{MLD}} & =\log\bar{\left(Y\right)}-\sum_{j=1}^{J}p_{j}\log\left(Y_{j}\right). \end{array}$$

#### 2.2.9. Theil’s Index (T)

T is similar to MLD but it uses a different disproportionality function. It is defined as

(34) $$\begin{array}{c} T=\sum_{j=1}^{J}p_{j}\gamma_{j}\log\left(\gamma_{j}\right), \end{array}$$

where $p_{j}$ and $\gamma_{j}$ are defined in (10) and (32), respectively. It is estimated by

(35) $$\begin{array}{c} \hat{T}=\sum_{j=1}^{J}\frac{p_{j}Y_{j}}{\bar{Y}}\log\left(\frac{Y_{j}}{\bar{Y}}\right). \end{array}$$

#### 2.2.10. Relative Index of Inequality (RII)

RII is obtained by dividing SII by the population mean rate [15]

(36) $$\begin{array}{c} \mathrm{RII}=\frac{\mathrm{SII}}{\bar{\mu }}=\frac{\beta_{1}}{\bar{\mu }}, \end{array}$$

where $\bar{\mu }$ and $\beta_{1}$ are defined in (11) and (26), respectively. It is estimated by

(37) $$\begin{array}{c} \hat{\mathrm{RII}}=\frac{1}{\sum_{j}\left(p_{j}z_{j}^{2}\right)-{\left(\sum_{j}\left(p_{j}z_{j}\right)\right)}^{2}}\left[\frac{\sum_{j}\left(p_{j}z_{j}Y_{j}\right)}{\bar{Y}}-\sum_{j}\left(p_{j}z_{j}\right)\right]. \end{array}$$

#### 2.2.11. Kunst–Mackenbach Relative Index (KMI)

Mackenbach and Kunst, 1997 [16] proposed an alternative to RII by dividing the rate of a hypothetical person with $z_{j}=0$ by the rate of a hypothetical person with $z_{j}=1$

(38) $$\begin{array}{c} \mathrm{KMI}=\frac{\beta_{0}}{\beta_{0}+\beta_{1}}, \end{array}$$

where $\beta_{0}$ and $\beta_{1}$ are defined in (26). It is estimated by

(39) $$\begin{array}{c} \hat{\mathrm{KMI}}=\frac{{\hat{\beta }}_{0}}{{\hat{\beta }}_{0}+\hat{\mathrm{SII}}}, \end{array}$$

where $\hat{\mathrm{SII}}$ is calculated in (27) and ${\hat{\beta }}_{0}$ can be obtained as

(40) $$\begin{array}{c} {\hat{\beta }}_{0}=\bar{Y}-\hat{\mathrm{SII}}\times \bar{z}, \end{array}$$

where $\bar{Y}$ is defined in (12) and

(41) $$\begin{array}{c} \bar{z}=\sum_{j=1}^{J}p_{j}z_{j}. \end{array}$$

### 2.3. Confidence Intervals Based on the Classical Method

The classical method used for variance estimation for the majority of the measures of health disparities implemented in HD*Calc is the Delta method. If $\hat{\theta }=F\left(Y\right)$ is an estimator of the true measure of health disparities θ, we approximate *F* by using a first-order Taylor series approximation around μ and then

$$\begin{array}{c} \mathrm{Var}\left(\hat{\theta }\right)\approx \mathrm{Var}\left\{\sum_{j=1}^{J}{\left(\frac{\partial F}{\partial y_{j}}\right)}_{y_{j}=\mu_{j}}\left(Y_{j}-\mu_{j}\right)\right\}, \end{array}$$

where $\mu =(\mu_{1},\ldots ,\mu_{J})$ is the mean of Y. Assuming that the *J* socioeconomic groups are independent, we obtain

(42) $$\begin{array}{c} \mathrm{Var}\left(\hat{\theta }\right)\approx \sum_{j=1}^{J}{\left(\frac{\partial F}{\partial y_{j}}\right)}_{y_{j}=\mu_{j}}^{2}\sigma_{j}^{2}, \end{array}$$

where $\sigma^{2}=\left(\sigma_{1}^{2},\ldots ,\sigma_{J}^{2}\right)$ is the main diagonal of the variance-covariance matrix of Y. We substitute the unknown parameters $\mu_{j}$ and $\sigma_{j}^{2}$ with their estimates to obtain $\hat{\mathrm{Var}}\left(\hat{\theta }\right)$, and then construct corresponding Wald confidence intervals for θ. Detailed derivations of the formulas for the estimated variances may be found in Ahn et al., 2018 [7] for 11 of the 15 measures of health disparities implemented in HD*Calc (all measures except eACI, eRCI, PD and PR) and on the HD*Calc website [4] for all 15 measures.

### 2.4. Fiducial Intervals

In this section, we describe new methods to construct fiducial intervals for measures of health disparities based on the use of approximate fiducial quantities. The fiducial inference is an approach to inference introduced by Fisher that has good frequentist properties [17,18].

#### 2.4.1. Fiducial Quantities (FQs)

Following Krishnamoorthy and Lee, 2010 [9], for an observed value $m_{jk}$ of the number of events $D_{jk}$, we have the equalities

(43) $$\begin{array}{c} \mathrm{Pr}\left(D_{jk}\geq m_{jk}|\lambda_{jk}\right)=\mathrm{Pr}\left(\frac{\chi_{2m_{jk}}^{2}}{2n_{jk}}<\lambda_{jk}|m_{jk}\right), \end{array}$$

and

(44) $$\begin{array}{c} \mathrm{Pr}\left(D_{jk}\leq m_{jk}|\lambda_{jk}\right)=\mathrm{Pr}\left(\frac{\chi_{2m_{jk}+2}^{2}}{2n_{jk}}>\lambda_{jk}|m_{jk}\right), \end{array}$$

where $\chi_{d}^{2}$ is a random variable following a chi-squared distribution with *d* degree of freedom. Garwood 1936 [19] proposed a related exact confidence interval for a Poisson mean

(45) $$\begin{array}{c} \left(\frac{1}{2n_{jk}}\chi_{2m_{jk};\alpha /2}^{2},\;\frac{1}{2n_{jk}}\chi_{2m_{jk}+2;1-\alpha /2}^{2}\right). \end{array}$$

Cox 1953 [20] introduced an approximate FQ for $\lambda_{jk}$, $\frac{\chi_{2m_{jk}+1}^{2}}{2n_{jk}}$. A related approximate fiducial interval is

(46) $$\begin{array}{c} \left(\frac{1}{2n_{jk}}\chi_{2m_{jk}+1;\alpha /2}^{2},\;\frac{1}{2n_{jk}}\chi_{2m_{jk}+1;1-\alpha /2}^{2}\right). \end{array}$$

Dempster 2008 [21] proposed another approximate FQ for $\lambda_{jk}$, a 50-50 mixture of $\frac{\chi_{2m_{jk}}^{2}}{2n_{jk}}$ and $\frac{\chi_{2m_{jk}+2}^{2}}{2n_{jk}}$.

An approximate FQ for a function of λs may be obtained by replacing the λs with their FQs in the function [18]. In our case, each measure of health disparities can be expressed as a function h(·) of $\lambda_{jk}$s, and an approximate FQ for

(47) $$\begin{array}{c} h(\lambda_{11},\ldots ,\lambda_{1K};\ldots ;\lambda_{J1},\ldots ,\lambda_{JK}), \end{array}$$

is obtained as

(48) $$\begin{array}{c} h({\hat{\lambda }}_{11},\ldots ,{\hat{\lambda }}_{1K};\ldots ;{\hat{\lambda }}_{J1},\ldots ,{\hat{\lambda }}_{JK}), \end{array}$$

where ${\hat{\lambda }}_{jk}$ is an approximate FQ of $\lambda_{jk}$.

#### 2.4.2. Simulation-Based Methods to Construct Fiducial Intervals

We use the above approximate FQs to construct three different fiducial intervals (FIs):

FI1. Simulate ${\hat{\lambda }}_{jk}$ from $\frac{\chi_{m_{jk}+1}^{2}}{2n_{jk}}$;
FI2. Simulate ${\hat{\lambda }}_{jk}$ from either $\frac{\chi_{m_{jk}}^{2}}{2n_{jk}}$ or $\frac{\chi_{m_{jk}+2}^{2}}{2n_{jk}}$, each with a 50% probability;
FI3. Simulate ${\hat{\lambda }}_{jk}$ from both $\frac{\chi_{m_{jk}}^{2}}{2n_{jk}}$ and $\frac{\chi_{m_{jk}+2}^{2}}{2n_{jk}}$.

For each method, we plug in the simulated ${\hat{\lambda }}_{jk}$s into the function h(·) to obtain the simulated values of the measures of health disparities $h({\hat{\lambda }}_{11},\ldots ,{\hat{\lambda }}_{1K};\ldots ;{\hat{\lambda }}_{J1},\ldots ,{\hat{\lambda }}_{JK})$. After performing *B* simulations, a 95% FI is constructed using the 2.5 and 97.5 percentiles of the set of simulated values for the measures of health disparities. For cells where no event is observed, i.e., $m_{jk}=0$, we follow Zhang et al. 2014 [22] and use

(49) $$\begin{array}{c} {\hat{\lambda }}_{jk}=\frac{1/n_{jk}}{n_{jk}+1}. \end{array}$$

### 2.5. Monte Carlo Simulation-Based Methods (MCS)

For the Monte Carlo simulation-based methods, we simulate values for the age-adjusted rates, $\mu_{j}$, instead of values for the cell rates $\lambda_{jk}$, as performed for the previously described fiducial methods, either from a truncated Normal distribution (MCS-N) or a Gamma distribution (MCS-G). The mean and the variance of the distribution from which we simulate values are the estimated mean and the estimated variance of the estimator of $\mu_{j}$. The use of these two distributions ensures that all simulated values are non-negative. When using the truncated Normal distribution, we simulate from a Normal distribution and discard the negative simulated values, i.e., keep only the non-negative simulated values. The adjustment (49) for zero counts is also applied. After we simulate values for ${\hat{\mu }}_{j}$, we use them to calculate the simulated values for the measures of health disparities. After performing *B* simulations, the 95% CI is constructed using the 2.5 and 97.5 percentiles of the set of simulated values for the measures of health disparities.

### 2.6. Simulation Study

We simulated data under nine different scenarios to allow different combinations of sample sizes and true rates per cell (where the cells are all cross-classifications of age groups and socioeconomic groups). For each scenario, we simulated data for the 12 cells that correspond to the combinations of three ordered socioeconomic groups and four age groups. Fixed weights, according to the WHO World Standard were applied to each age group. Table 1 describes the characteristics of the nine scenarios, with the means and standard deviations (SDs) being calculated across the 12 cells. The combinations of sample sizes and true rates per cell resulted in five categories for the magnitude of the expected count per cell, i.e., <1, 1–9, 10–99, 100–999, and 1000–9999.

For each scenario, we generated 5000 datasets, and for each dataset, we used 5000 simulations to construct the 95% MCS-based CIs and the 95% FIs. The empirical coverage was defined as the frequency of the true value of the measure of health disparities being covered by the nominal 95% CIs or FIs.

## 3. Results

**Figure 1:**
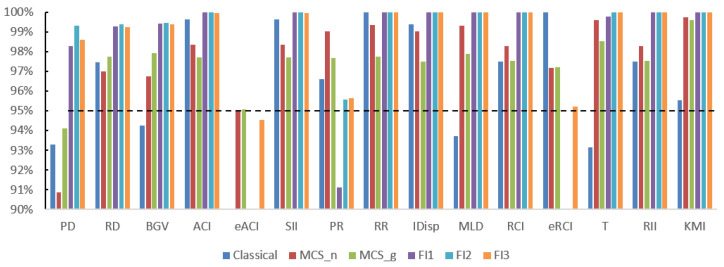
Empirical coverage results for scenario 1. The dashed line shows 95% coverage.

We start with the results for scenario 1, which corresponds to a situation involving extremely sparse data, i.e., where the expected count per cell is below 1. The empirical coverage results are presented in Table 2 and Figure 1. For eACI, the Classic method, FI1 and FI2 had empirical coverages considerably below the nominal 95% level; FI1 and FI2 had the same problem for eRCI. The MCS-N method had only about 91% empirical coverage for PD, while FI3 had very large empirical coverage ranging from 99% to 100% for 11 of the 15 measures. By contrast, the MCS-G method performed reasonably well for all 15 measures for this scenario.

**Figure 2:**
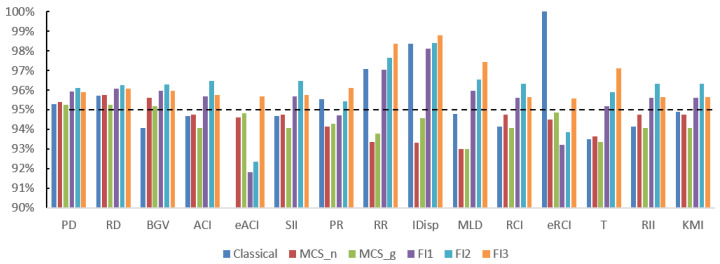
Empirical coverage results for scenario 2. The dashed line shows 95% coverage.

**Figure 3:**
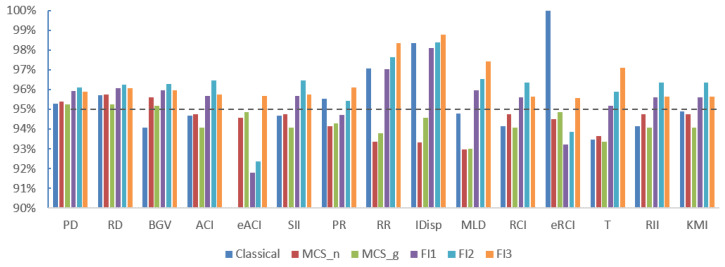
Empirical coverage results for scenario 3. The dashed line shows 95% coverage.

The results for scenarios 2 and 3 were very similar to each other. They both correspond to situations involving sparse (but not extremely sparse) data, where the expected count per cell is between 1 and 10. The empirical coverage results are shown in Table 3 and Figure 2 for scenario 2, and in Table 4 and Figure 3 for scenario 3, respectively. For both scenarios, the Classic method still had empirical coverages considerably below the nominal 95% level for eACI. FI1 and FI2 had the same problem for eACI, but to a much lesser extent, with empirical coverages of about 92%. Overall, FI3 performed best for the 15 measures, followed closely by MCS-G and MCS-N.

For scenarios 4 to 9, where the data may not be considered sparse by having an expected count per cell of 10 or more, the empirical coverages are between 94% and 96% for all methods and all 15 measures, except for the Classical method for eACI (where they ranged from 79% to 83%) and eRCI (where they were all 100%). For these scenarios, all methods except the Classical method performed well. The complete results regarding the empirical coverage are shown in Appendix A.

## 4. Discussion

We compared six methods to construct confidence intervals and fiducial intervals for 15 measures of health disparities with regard to their empirical coverage under nine different scenarios. Overall, two methods performed well: the MCS-G method to construct confidence intervals and the FI3 method to construct fiducial intervals. It is important to note that the documentation for HD*Calc version 2.0.0 also recommends the use of the Monte Carlo simulation-based method with the Gamma distribution based on the results from Ahn et al., 2019 [8] regarding 11 measures of health disparities. Compared to the Normal distribution, the Gamma distribution is a better choice to use for simulating rates due to its positivity. Moreover, its flexibility in accommodating asymmetry surpasses that of a truncated Normal distribution.

The strengths of the current study include the addition of four measures (eACI, eRCI, PD and PR) to the list of 11 measures of health disparities previously investigated, and the consideration of different scenarios corresponding to different combinations of sample sizes and true rates per cell. The limitations, due to feasibility reasons, include the consideration of eACI and eRCI only when the aversion parameter ν=3, the use of only one value for the number of simulations used for the MCS-based methods and the fiducial methods, i.e., 5000 simulations, the use of an ordinal socioeconomic group variable with only three levels, and the use of an age group variable with only four levels.

Future research work should consider eACI and eRCI with other values of the aversion parameter, a larger number of socioeconomic groups and age groups, and different numbers of simulations for the MCS-based methods and the fiducial methods. Building upon the work from Talih et al., 2020 [23], related future research should also investigate if it is possible to reduce a large number of measures of health disparities to a smaller set of measures that satisfy a set of desirable properties and are easier to interpret. With a smaller number of recommended measures of health disparities, it would be easier to thoroughly compare the performance of statistical methods to construct confidence intervals and fiducial intervals for these selected measures.

## 5. Conclusions

Given that the MCS-G method is much simpler to understand and implement than the FI3 method, and the lack of familiarity of statisticians and (more importantly) practitioners with fiducial methods and fiducial intervals, we recommend the use of the Monte Carlo simulation-based method with the Gamma distribution.

## Figures and Tables

**Table 1:** Characteristics of the nine scenarios.

Scenario	Sample Size Mean (SD)	True Rate Mean (SD)	Expected Event Count Mean (SD)	Magnitude of Expected Event Count
1	2417 (1084)	0.0003 (0.0002)	0.8 (0.784)	<1
2	2417 (1084)	0.003 (0.002)	8 (7.84)	1–9
3	24,167 (10,836)	0.0003 (0.0002)	8 (7.84)	1–9
4	2417 (1084)	0.03 (0.02)	80 (78.4)	10–99
5	24,167 (10,836)	0.003 (0.002)	80 (78.4)	10–99
6	241,667 (108,363)	0.0003 (0.0002)	80 (78.4)	10–99
7	24,167 (10,836)	0.03 (0.02)	800 (784)	100–999
8	241,667 (108,363)	0.003 (0.002)	800 (784)	100–999
9	241,667 (108,363)	0.03 (0.02)	8000 (7839)	1000–9999

**Table 2:** Empirical coverage (%) for nominal 95% confidence intervals and fiducial intervals for scenario 1.

Measure	Classical	MCS-N	MCS-G	FI1	FI2	FI3
RD	97.46	97.00	97.74	99.30	99.38	99.26
PD	93.28	90.86	94.12	98.30	99.32	98.60
BGV	94.26	96.74	97.94	99.42	99.46	99.38
ACI	99.64	98.34	97.72	99.98	100.0	99.96
eACI	82.82	95.04	95.08	52.20	64.10	94.52
SII	99.64	98.34	97.72	99.98	100.0	99.96
RR	100.0	99.36	97.76	100.0	100.0	100.0
PR	96.62	99.04	97.66	91.12	95.56	95.64
IDisp	99.40	99.04	97.50	100.0	100.0	100.0
MLD	93.72	99.32	97.88	99.98	100.0	100.0
RCI	97.50	98.30	97.54	99.98	100.0	100.0
eRCI	100.0	97.16	97.22	71.58	80.94	95.20
T	93.14	99.60	98.54	99.78	99.98	99.98
RII	97.50	98.30	97.54	99.98	100.0	100.0
KMI	95.54	99.76	99.62	99.98	100.0	100.0

**Table 3:** Empirical coverage (%) for nominal 95% confidence intervals and fiducial intervals for scenario 2.

Measure	Classical	MCS-N	MCS-G	FI1	FI2	FI3
RD	95.70	95.76	95.26	96.08	96.24	96.06
PD	95.28	95.38	95.26	95.92	96.10	95.90
BGV	94.08	95.60	95.18	95.98	96.28	95.98
ACI	94.68	94.76	94.06	95.68	96.48	95.74
eACI	78.94	94.60	94.82	91.80	92.36	95.68
SII	94.68	94.76	94.06	95.68	96.48	95.74
RR	97.08	93.34	93.80	97.04	97.64	98.36
PR	95.52	94.14	94.30	94.72	95.42	96.10
IDisp	98.36	93.32	94.58	98.10	98.40	98.78
MLD	94.80	92.98	93.00	95.98	96.54	97.44
RCI	94.14	94.76	94.06	95.62	96.34	95.64
eRCI	100.0	94.50	94.84	93.22	93.86	95.58
T	93.48	93.64	93.36	95.16	95.90	97.10
RII	94.14	94.76	94.06	95.62	96.34	95.64
KMI	94.90	94.76	94.06	95.62	96.34	95.64

**Table 4:** Empirical coverage (%) for nominal 95% confidence intervals and fiducial intervals for scenario 3.

Measure	Classical	MCS-N	MCS-G	FI1	FI2	FI3
RD	95.70	95.76	95.26	96.08	96.24	96.06
PD	95.28	95.38	95.26	95.92	96.10	95.90
BGV	94.08	95.60	95.18	95.98	96.28	95.98
ACI	94.68	94.76	94.06	95.68	96.48	95.74
eACI	78.70	94.58	94.84	91.80	92.34	95.66
SII	94.68	94.76	94.06	95.68	96.48	95.74
RR	97.08	93.34	93.80	97.04	97.64	98.36
PR	95.52	94.14	94.30	94.72	95.42	96.10
IDisp	98.36	93.32	94.58	98.10	98.40	98.78
MLD	94.80	92.98	93.00	95.98	96.54	97.44
RCI	94.14	94.76	94.06	95.62	96.34	95.64
eRCI	100.0	94.50	94.84	93.22	93.86	95.58
T	93.48	93.64	93.36	95.16	95.90	97.10
RII	94.14	94.76	94.06	95.62	96.34	95.64
KMI	94.90	94.76	94.06	95.62	96.34	95.64

## Data Availability

The code used to simulate the data for this study is available upon request from the corresponding author.

## Abbreviations

The following abbreviations are used in this manuscript:

ACI	absolute concentration index
BGV	between-group variance
CDC	Centers for Disease Control and Prevention
CI	confidence interval
eACI	extended absolute concentration index
eRCI	extended relative concentration index
FI	fiducial interval
FQ	fiducial quantity

HD*Calc	Health Disparity Calculator
IDisp	index of disparity
KMI	Kunst-Mackenbach relative index
MCS	Monte Carlo simulation
MCS-N	Monte Carlo simulation-based using a truncated Normal distribution
MCS-G	Monte Carlo simulation-based using the Gamma distribution
MLD	mean log deviation
PD	pair difference
PR	pair ratio
RCI	relative concentration index
RD	range difference
RII	relative index of inequality
RR	range ratio
SEER	Surveillance, Epidemiology, and End Results
SII	slope index of inequality
T	Theil’s index
WHO	World Health Organization

## Appendix A. Results of the Simulation Study

**Table A1:** Empirical coverage (%) for nominal 95% confidence intervals and fiducial intervals for all scenarios.

Measure	Scenario	Classical	MCS-N	MCS-G	FI1	FI2	FI3
PD	1	93.28	90.86	94.12	98.30	99.32	98.60
	2	95.28	95.38	95.26	95.92	96.10	95.90
	3	95.28	95.38	95.26	95.92	96.10	95.90
	4	95.28	95.32	95.40	95.40	95.20	95.30
	5	95.28	95.32	95.40	95.40	95.20	95.30
	6	95.28	95.32	95.40	95.40	95.20	95.30
	7	94.66	94.74	94.78	94.74	94.76	94.72
	8	94.66	94.74	94.78	94.74	94.76	94.72
	9	94.80	94.78	94.80	94.78	94.78	94.92
RD	1	97.46	97.00	97.74	99.30	99.38	99.26
	2	95.70	95.76	95.26	96.08	96.24	96.06
	3	95.70	95.76	95.26	96.08	96.24	96.06
	4	95.28	95.36	95.40	95.40	95.18	95.28
	5	95.28	95.36	95.40	95.40	95.18	95.28
	6	95.28	95.36	95.40	95.40	95.18	95.28
	7	94.66	94.74	94.78	94.74	94.76	94.72
	8	94.66	94.74	94.78	94.74	94.76	94.72
	9	94.80	94.78	94.80	94.78	94.78	94.92
BGV	1	94.26	96.74	97.94	99.42	99.46	99.38
	2	94.08	95.60	95.18	95.98	96.28	95.98
	3	94.08	95.60	95.18	95.98	96.28	95.98
	4	95.06	95.06	95.12	95.10	95.12	95.08
	5	95.06	95.06	95.12	95.10	95.12	95.08
	6	95.06	95.06	95.12	95.10	95.12	95.08
	7	95.24	95.40	95.18	95.36	95.30	95.34
	8	95.24	95.40	95.18	95.36	95.30	95.34
	9	94.80	94.76	94.88	94.86	94.84	94.82
ACI	1	99.64	98.34	97.72	99.98	100.0	99.96
	2	94.68	94.76	94.06	95.68	96.48	95.74
	3	94.68	94.76	94.06	95.68	96.48	95.74
	4	95.40	95.48	95.12	95.40	95.56	95.40
	5	95.40	95.48	95.12	95.40	95.56	95.40
	6	95.40	95.48	95.12	95.40	95.56	95.40
	7	94.54	94.48	94.54	94.68	94.42	94.54
	8	94.54	94.48	94.54	94.68	94.42	94.54
	9	94.62	94.70	94.72	94.60	94.62	94.64
eACI	1	82.82	95.04	95.08	52.20	64.10	94.52
	2	78.94	94.60	94.82	91.80	92.36	95.68
	3	78.70	94.58	94.84	91.80	92.34	95.66
	4	81.46	94.42	94.60	94.10	94.20	94.40
	5	79.38	94.50	94.62	94.06	94.10	94.40
	6	79.24	94.52	94.62	94.04	94.12	94.40
	7	81.78	95.28	95.20	95.24	95.36	95.26
	8	80.14	95.22	95.20	95.24	95.32	95.30
	9	83.10	95.54	95.54	95.56	95.52	95.46
SII	1	99.64	98.34	97.72	99.98	100.0	99.96
	2	94.68	94.76	94.06	95.68	96.48	95.74
	3	94.68	94.76	94.06	95.68	96.48	95.74
	4	95.40	95.48	95.12	95.40	95.56	95.40
	5	95.40	95.48	95.12	95.40	95.56	95.40
	6	95.40	95.48	95.12	95.40	95.56	95.40
	7	94.54	94.48	94.54	94.68	94.42	94.54
	8	94.54	94.48	94.54	94.68	94.42	94.54
	9	94.62	94.70	94.72	94.60	94.62	94.64
PR	1	96.62	99.04	97.66	91.12	95.56	95.64
	2	95.52	94.14	94.30	94.72	95.42	96.10
	3	95.52	94.14	94.30	94.72	95.42	96.10
	4	95.66	95.64	95.44	95.36	95.62	95.62
	5	95.66	95.64	95.44	95.36	95.62	95.62
	6	95.66	95.64	95.44	95.36	95.62	95.62
	7	94.80	94.88	94.80	94.68	94.80	94.84
	8	94.80	94.88	94.80	94.68	94.80	94.84
	9	94.94	94.84	94.82	94.92	94.98	94.98
RR	1	100.0	99.36	97.76	100.0	100.0	100.0
	2	97.08	93.34	93.80	97.04	97.64	98.36
	3	97.08	93.34	93.80	97.04	97.64	98.36
	4	95.68	95.70	95.54	95.44	95.70	95.68
	5	95.68	95.70	95.54	95.44	95.70	95.68
	6	95.68	95.70	95.54	95.44	95.70	95.68
	7	94.80	94.88	94.80	94.68	94.80	94.84
	8	94.80	94.88	94.80	94.68	94.80	94.84
	9	94.94	94.84	94.82	94.92	94.98	94.98
IDisp	1	99.40	99.04	97.50	100.0	100.0	100.0
	2	98.36	93.32	94.58	98.10	98.40	98.78
	3	98.36	93.32	94.58	98.10	98.40	98.78
	4	95.14	95.58	95.42	95.32	95.48	95.56
	5	95.14	95.58	95.42	95.32	95.48	95.56
	6	95.14	95.58	95.42	95.32	95.48	95.56
	7	94.80	94.44	94.64	94.70	94.58	94.68
	8	94.80	94.44	94.64	94.70	94.58	94.68
	9	94.92	94.88	94.82	94.86	94.88	94.96
MLD	1	93.72	99.32	97.88	99.98	100.0	100.0
	2	94.80	92.98	93.00	95.98	96.54	97.44
	3	94.80	92.98	93.00	95.98	96.54	97.44
	4	95.70	95.06	95.10	95.74	95.82	96.06
	5	95.70	95.06	95.10	95.74	95.82	96.06
	6	95.70	95.06	95.10	95.74	95.82	96.06
	7	95.02	95.14	95.02	94.98	95.00	95.06
	8	95.02	95.14	95.02	94.98	95.00	95.06
	9	94.84	94.78	94.92	94.70	94.84	94.80
RCI	1	97.50	98.30	97.54	99.98	100.0	100.0
	2	94.14	94.76	94.06	95.62	96.34	95.64
	3	94.14	94.76	94.06	95.62	96.34	95.64
	4	95.28	95.46	95.04	95.22	95.56	95.34
	5	95.28	95.46	95.04	95.22	95.56	95.34
	6	95.28	95.46	95.04	95.22	95.56	95.34
	7	94.60	94.62	94.64	94.62	94.64	94.70
	8	94.60	94.62	94.64	94.62	94.64	94.70
	9	94.90	94.86	94.88	94.78	94.84	94.82
eRCI	1	100.0	97.16	97.22	71.58	80.94	95.20
	2	100.0	94.50	94.84	93.22	93.86	95.58
	3	100.0	94.50	94.84	93.22	93.86	95.58
	4	100.0	95.02	95.14	95.08	95.10	95.46
	5	100.0	95.02	95.14	95.08	95.10	95.46
	6	100.0	95.02	95.14	95.08	95.10	95.46
	7	100.0	94.82	94.76	94.80	94.86	94.86
	8	100.0	94.82	94.76	94.80	94.86	94.86
	9	100.0	95.38	95.40	95.38	95.12	95.28
T	1	93.14	99.60	98.54	99.78	99.98	99.98
	2	93.48	93.64	93.36	95.16	95.90	97.10
	3	93.48	93.64	93.36	95.16	95.90	97.10
	4	95.52	95.12	95.20	95.64	95.70	95.92
	5	95.52	95.12	95.20	95.64	95.70	95.92
	6	95.52	95.12	95.20	95.64	95.70	95.92
	7	95.20	95.06	95.06	95.16	95.16	95.16
	8	95.20	95.06	95.06	95.16	95.16	95.16
	9	94.88	94.82	94.92	94.78	94.76	94.84
RII	1	97.50	98.30	97.54	99.98	100.0	100.0
	2	94.14	94.76	94.06	95.62	96.34	95.64
	3	94.14	94.76	94.06	95.62	96.34	95.64
	4	95.28	95.46	95.04	95.22	95.56	95.34
	5	95.28	95.46	95.04	95.22	95.56	95.34
	6	95.28	95.46	95.04	95.22	95.56	95.34
	7	94.60	94.62	94.64	94.62	94.64	94.70
	8	94.60	94.62	94.64	94.62	94.64	94.70
	9	94.90	94.86	94.88	94.78	94.84	94.82
KMI	1	95.54	99.76	99.62	99.98	100.0	100.0
	2	94.90	94.76	94.06	95.62	96.34	95.64
	3	94.90	94.76	94.06	95.62	96.34	95.64
	4	95.18	95.46	95.04	95.22	95.56	95.34
	5	95.18	95.46	95.04	95.22	95.56	95.34
	6	95.18	95.46	95.04	95.22	95.56	95.34
	7	94.66	94.62	94.64	94.62	94.64	94.70
	8	94.66	94.62	94.64	94.62	94.64	94.70
	9	94.84	94.86	94.88	94.78	94.84	94.82
